# Weight bias internalization in a commercial weight management sample: prevalence and correlates

**DOI:** 10.1002/osp4.354

**Published:** 2019-07-11

**Authors:** R. L. Pearl, M. S. Himmelstein, R. M. Puhl, T. A. Wadden, A. C. Wojtanowski, G. D. Foster

**Affiliations:** ^1^ Center for Weight and Eating Disorders, Department of Psychiatry Perelman School of Medicine at the University of Pennsylvania Philadelphia PA USA; ^2^ Rudd Center for Food Policy and Obesity University of Connecticut Hartford CT USA; ^3^ Department of Psychological Sciences Kent State University Kent OH USA; ^4^ Department of Human Development and Family Sciences University of Connecticut, Storrs Storrs CT USA; ^5^ WW New York NY USA

**Keywords:** Psychosocial variables, stigma, weight management programme

## Abstract

**Objective:**

Weight bias internalization (WBI) is associated with poor weight‐related health. The purpose of this study was to identify the prevalence and correlates of WBI in a large sample of adults in a commercial weight management programme.

**Methods:**

WW (the new Weight Watchers) members participated in an online survey. Participants (*N* = 18,769) completed the 10‐item Weight Bias Internalization Scale – Modified (WBIS‐M) and the Weight Self‐Stigma Questionnaire (WSSQ). Participants reported details about weight‐stigmatizing experiences, including the onset, frequency and distress, and interpersonal sources of weight stigma. Participants self‐reported their demographics, weight history, and height and weight (to compute body mass index [BMI]).

**Results:**

Weight bias internalization was relatively high compared with the general population (mean WBIS‐M score = 4.3 ± 1.4; mean WSSQ total score = 35.2 ± 9.7). WBI was higher among participants who were female, younger and had higher BMIs (*p* < 0.001) and lower among those who were Black and were widowed or had a romantic partner (*p* < 0.001). Onset of weight stigma in childhood and young adulthood, and recent distress due to weight stigma, predicted higher WBI. Extended family and school sources of weight stigma had weaker associations with WBI than did other interpersonal sources.

**Conclusions:**

Weight bias was internalized by a significant proportion of adults enrolled in a commercial weight management programme. A phenotype of WBI includes demographic characteristics and the timing and sources of weight stigma.

## Introduction

Weight bias internalization (WBI) – also known as self‐directed weight stigma – occurs when individuals with overweight/obesity become aware of negative weight‐based stereotypes and apply those stereotypes to themselves 1. Thus, they turn weight‐based societal scorn and devaluation inward and onto themselves 1, 2. WBI is a relatively new construct that has received increasing attention in the obesity field because of its adverse implications for health 3. Prior studies investigating WBI have primarily drawn from small community or treatment‐seeking samples 1, 3, 4, 5, 6, 7, with the exception of a few large population‐based studies 8, 9, 10. Across these studies, some demographic groups (e.g. women and White adults) have consistently reported elevated levels of WBI 3. However, relationships between WBI and other characteristics, such as age, education and body mass index (BMI), have been inconsistent 3, 8, 9, 10, 11, 12. Additionally, little is known about psychosocial correlates of WBI, such as specific types of weight‐stigmatizing experiences (e.g. from family at a young age versus in the workplace at an older age) 13. A more clearly defined phenotype of who internalizes weight bias – based on demographic characteristics and information about when, by whom, and to what degree individuals are affected by weight‐stigmatizing experiences – is needed to further develop WBI prevention and intervention efforts.

In recent years, more evidence of WBI's negative relationship to weight‐related health behaviours has emerged 3. For example, WBI has been associated with increased binge eating in 18 studies, reduced physical activity engagement and motivation in 10 studies and reduced self‐monitoring and other effective weight‐control behaviours in four studies 3. Two recent studies have also suggested that WBI predicts less long‐term maintenance of lost weight 14, 15. Despite this evidence, WBI is rarely addressed in most weight management programmes 3, 16. A comprehensive evaluation of WBI in adults seeking weight‐loss treatment is necessary to better identify individuals at risk for WBI and prevent downstream impaired behavioural and weight‐loss outcomes.

Improving current knowledge of the nature and extent of WBI among treatment‐seeking individuals requires more attention to measurement of this construct. Variation in measurement of WBI may account for some inconsistencies in previous findings and presents a challenge for systematically comparing effects across studies 3. The primary measures of WBI are the Weight Bias Internalization Scale (WBIS) 1, the weight‐neutral version of the scale (WBIS – Modified [M]) 17, and the Weight Self‐Stigma Questionnaire (WSSQ; including a total score and scores on Fear of Enacted Stigma [FES] and Self‐Devaluation [SD] subscales) 6. Because of differing psychometrics, studies have varied in how many items from the WBIS and WBIS‐M are included 3. The WSSQ‐FES subscale also diverges from some definitions of internalized stigma by including items pertaining to anticipated stigma, which may be a separate (though related) construct 18. Greater examination and comparison of measures – their content and their correlates – will help to improve the rigor and consistency of research on WBI.

The current study had three aims: (1) to determine the extent to which adults engaged in weight loss reported WBI; (2) to identify correlates of WBI, including demographic characteristics and specific types of weight‐stigmatizing experiences; and (3) to compare these correlates between WBI measures (WBIS‐M versus WSSQ). These aims were evaluated in a large sample of adults enrolled in WW (the new Weight Watchers), an empirically validated behavioural weight management programme that encourages healthy habits related to food, activity and mindset 19, 20, 21, 22. Given that millions of Americans participate in weight management programmes each year 23, these treatment‐seeking adults may represent a more generalizable sample than those from prior smaller, more tightly controlled treatment efficacy studies in clinical settings.

## Methods

The study was open to WW members residing in the USA who were 18 years or older and had participated in WW for at least 3 months. Data were collected from September 2017 to April 2018 from ‘Digital + Studio’ WW members, whose membership included in‐person workshops (i.e., studio) and access to the WW app and online tools (i.e., digital). Members were recruited via an email from WW inviting them to participate. For the first 2 weeks, invitation emails were sent to a random set of 10,000 Digital + Studio members; for the remaining weeks, this was increased to 15,000 per week (totalling 305,000 Digital + Studio members). Additionally, a random sample of Digital members who had access to the WW app and online tools – but did not attend in‐person workshops – and met the inclusion criteria earlier was recruited via invitation emails sent by WW between February and August 2018 (15,000 per week in the first month, 25,000 per week for the following 3 months and 35,000 per week for the final 3 months, totalling 850,000 Digital members).

### Procedures

The random sample of WW members received an email describing an anonymous, voluntary online survey pertaining to ‘experiences related to body weight and health, and challenges that come with these experiences such as stress, self‐confidence and, stigma’. Interested participants could click an anonymous link to the survey (managed by the study researchers and hosted by Qualtrics), which began with an informed consent form. After clicking to provide consent, participants entered the survey consisting of an online battery of measures. The measures presented in this paper were part of a larger study examining weight stigma and its correlates with weight‐related health behaviours. This study received institutional review board approval.

### Measures

#### Weight bias internalization

The 10‐item version of the WBIS‐M, as well as the WSSQ (total score and two subscales), were included in the survey. Prior studies have suggested that removing the first item of the original 11‐item WBIS improves its psychometric properties 24, 25, and the WBIS‐M allows for assessment of WBI among individuals who do not identify as having overweight/obesity 17. Scale items assess weight‐related stereotype endorsement (e.g., ‘I am less attractive than most other people because of my weight’) and self‐devaluation due to weight (e.g., ‘I hate myself for my weight’). WBIS‐M scores are computed by averaging the 10 items (rated on a 1–7 scale), with higher scores indicating greater WBI. In the current sample, the scale had strong internal consistency (Cronbach's *α* = 0.91). In addition to mean WBIS‐M scores, prevalence of WBI was computed for this study with the frequency of participants scoring ≥4.0 and ≥4.88 on the WBIS‐M. These cut‐offs have been used in prior research to indicate ‘high’ WBI based on the midpoint of the scale 26 and one standard deviation above population norms 8.

The WSSQ consists of 12 items, divided into the two subscales of FES and SD (six items each) 6. FES items assess perceived and anticipated stigma from others (e.g., ‘Others will think I lack self‐control because of my weight problems’), and SD items assess negative self‐perceptions and self‐blame due to weight (e.g., ‘I became overweight because I'm a weak person’). Items are rated from 1 to 5 and summed, with higher scores indicating greater WBI. The internal consistency of the total scale and each subscale was strong (*α* = 0.89, 0.88 and 0.81, respectively). No cut‐offs have been established for WSSQ scores.

#### Weight stigma experiences

Participants responded to three yes/no items used in prior research asking if they had ever been teased, treated unfairly, or discriminated against because of their weight 27. A dichotomous variable was computed to indicate whether or not participants endorsed any of these three items (i.e., had or had not experienced weight stigma).

#### Weight Stigma Time of Life Questionnaire (WSTOLQ)

The WSTOLQ was developed for the current study and administered only to participants who reported experiencing weight stigma. Participants were asked to indicate the time period in which they first experienced weight stigma (i.e., onset): childhood (≤10 years), adolescence (11–19 years), young adulthood (20–39 years), middle adulthood (40–59 years) or older adulthood (≥60 years). Participants also rated (1–7) the frequency (‘never’ to ‘extremely often’) and distress (‘not at all upset’ to ‘extremely upset’) of their weight‐stigmatizing experiences during each time period and in the last year.

#### Interpersonal sources of weight stigma

Developed and tested in prior research 13, 28, the Interpersonal Sources of Stigma scale assesses the frequency (never, once in your life, more than once in your life, or multiple times) participants had experienced weight stigma from 25 potential people. Individual sources were grouped into the following categories: family of origin (mother, father, sister, brother), extended family (grandmother, grandfather, aunt, uncle, cousin), family of procreation (spouse, son, daughter), friends, workplace (coworker, employer/supervisor), school (classmate, teacher/professor), health care (doctor, nurse, dietitian/nutritionist, mental health professional) and community (authority figure, general community members, sales clerk, restaurant servers). For this study, source categories were coded as ‘yes’ if participants reported experiencing weight stigma once in their life from any of the included individual source items. Participants who did not encounter a given interpersonal source (e.g., did not have a brother) could respond ‘never’ or leave the item blank.

#### Participant characteristics

Participants reported their sex, race/ethnicity, age, marital status and highest level of education. BMI was computed from self‐reported height and weight, and participants were divided into BMI categories. Participants reported their age of overweight onset, duration of their WW membership (3–6 months, 6–12 months, 1–5 years, ≥6 years) and WW membership type (Digital + Studio versus Digital).

### Statistical analyses

Descriptive statistics were computed for all variables. Correlations and analyses of variance (ANOVAs) were used to identify participant characteristics associated with WBIS‐M and WSSQ total scores. *Post hoc* pairwise comparisons were used to identify differences in WBI within categories of variables (e.g., race/ethnicity).

Separate regression models, controlling for all participant characteristics with independent associations with WBI, were conducted to test the effects on WBIS‐M and WSSQ total scores of: experiences of weight stigma (all three items and yes/no to any); time period of weight stigma onset; frequency and distress of weight‐stigmatizing experiences during each time period and in the last year; and interpersonal sources of weight stigma. All weight stigma variables were initially tested in separate regression models to establish independent relationships with WBI. The exceptions were frequency and distress of stigma variables for the same time periods (e.g., childhood), which were included in the same models in order to assess relative predictive strength. Additional tests of models, including all variables within each variable category (e.g., all interpersonal sources of stigma), were conducted to identify the total amount of variance in WBI accounted for by the construct. Supplemental analyses were conducted repeating all analyses for the two WSSQ subscales.

Because of the number of analyses and large sample size, only *p* values ≤0.001 were considered significant 29, 30.

## Results

### Participants

A total of 25,967 individuals entered the survey website (3.5% response rate for Digital + Studio members and 0.8% response rate for Digital members). Of those, 2,535 participants were ineligible for the study because they declined to consent (*n* = 658), had a WW membership for less than 3 months (*n* = 477), were under the age of 18 (*n* = 17), closed the survey before completing the eligibility questions (*n* = 1,290) or indicated only participating in the digital WW programming before the study expanded to include Digital members (i.e., before February of 2018; *n* = 93). A total of 23,432 individuals who entered the survey were eligible to complete it; 4,663 were excluded for failing to complete at least 50% of the survey (*n* = 2,728) or for failing to provide key demographic or anthropometric information (i.e. BMI, sex, race: *n* = 1,935). After all exclusions, the final sample consisted of 18,769 participants.

Table 1 reports participant characteristics for the full sample and separately for Digital + Studio members (56.5%) and Digital members (43.5%). Some differences emerged between member groups. For example, relative to Digital members, Digital + Studio members were older (M = 54.8 vs. 48.8 years, *p* < 0.001) and had a higher mean BMI (32.3 vs. 31.4 kg m^−2^, *p* < 0.001).

**Table 1:** Sample characteristics: mean ± standard deviation or n (%)

Variable	Total sample (N = 18,769)	Digital + Studio members (N = 10,606)	Digital members (N = 8,163)	p value
Age	52.2 ± 12.9	54.8 ± 12.2	48.8 ± 12.9	<0.001
Sex				0.001
Male	1,006 (5.4)	517 (4.9)	489 (6.0)	
Female	17,763 (94.6)	10,089 (95.1)	7,674 (94.0)	
Race/Ethnicity				0.006
White, non‐Hispanic/Latino	17,095 (91.1)	9,702 (91.5)	7,393 (90.6)	
Black or African–American	592 (3.2)	325 (3.1)	267 (3.3)	
Asian or Pacific Islander	117 (0.6)	48 (0.5)	69 (0.8)	
Latino, Hispanic or Mexican–American	641 (3.4)	345 (3.3)	296 (3.6)	
Other	324 (1.7)	186 (1.8)	138 (1.7)	
Education				<0.001
Less than high school/GED	24 (0.1)	15 (0.1)	9 (0.1)	
High school/GED	1,239 (6.6)	770 (7.3)	469 (5.7)	
Vocational/technical school (2 years)	899 (4.8)	552 (5.2)	347 (4.3)	
Some college	3,580 (19.1)	2,226 (21.0)	1,354 (16.6)	
College graduate	6,849 (36.5)	3,839 (36.2)	3,010 (36.9)	
Postgraduate degree or higher	6,178 (32.9)	3,204 (30.2)	2,974 (36.4)	
Marital status				<0.001
Married	13,242 (70.6)	7,483 (70.6)	5,759 (70.6)	
Divorced	1,938 (10.3)	1,159 (10.9)	779 (9.5)	
Separated	132 (0.7)	73 (0.7)	59 (0.7)	
Widowed	723 (3.9)	488 (4.6)	235 (2.9)	
Never married	2,710 (14.4)	1,388 (13.1)	1,322 (16.2)	
Current significant other				<0.001
Yes	15,129 (80.6)	8,396 (79.2)	6,733 (82.5)	
No	3,569 (19.0)	2,167 (20.4)	1,402 (17.2)	
BMI	31.9 ± 7.0	32.3 ± 6.9	31.4 ± 7.1	<0.001
BMI category (kg m^−2^)				<0.001
<18.5	26 (0.1)	11 (0.1)	15 (0.2)	
18.5–24.9	2,418 (12.9)	1,038 (9.8)	1,380 (16.9)	
25–29.9	6,283 (33.5)	3,629 (34.2)	2,654 (32.5)	
≥30	10,042 (53.5)	5,928 (55.9)	4,114 (46.8)	
Age of onset of overweight	22.4 ± 12.5	22.4 ± 12.8	22.3 ± 12.3	0.869
Time in WW				<0.001
3–6 months	3,086 (16.4)	1,842 (17.4)	1,244 (15.2)	
6–12 months	5,729 (30.5)	3,217 (30.3)	2,512 (30.8)	
1–5 years	7,833 (41.7)	4,128 (38.9)	3,705 (45.4)	
6+ years	2,121 (11.3)	1,419 (13.4)	702 (8.6)	

Digital + Studio members attended in‐person workshops and had access to the WW app and online tools. Because of missing data, *n*s for total sample ranged from 18,531 to 18,769, *n*s for Digital + Studio members ranged from 10,448 to 10,606 and *n*s for Digital members ranged from 8,083 to 8,163. Analyses of variance and chi‐squared tests were used to identify differences in participant characteristics between Digital + Studio and Digital members.

BMI, body mass index; GED, General Education Development.

### Prevalence of weight bias internalization and experiences

Table 2 presents all means and frequencies for WBI and weight stigma variables. Mean WBI scores were relatively high across measures. For example, 58.3% of the sample had a WBIS‐M score ≥4.0, and 35.5% scored ≥4.88. In total, 63.5% of participants reported having had an experience of weight stigma: 58.7% reported teasing; 34.2% reported discrimination; and 43.7% reported unfair treatment due to weight. Almost half (46.3%) of participants reported that they first experienced weight stigma during childhood or adolescence. Frequency and distress related to weight stigma were also higher during childhood and adolescence compared with other time periods. Approximately 40–50% of participants reported experiencing weight stigma from each interpersonal source.

**Table 2:** Weight stigma characteristics: mean ± standard deviation or n (%)

Variable	Total sample	Digital + Studio members	Digital members
Weight Bias Internalization Scale – Modified	4.3 ± 1.4	4.2 ± 1.4	4.3 ± 1.4
Weight Self‐Stigma Questionnaire	35.2 ± 9.7	35.2 ± 9.6	35.2 ± 9.8
Fear of Enacted Stigma Subscale	17.8 ± 5.7	18.0 ± 5.7	17.6 ± 5.8
Self‐Devaluation Subscale	17.4 ± 5.0	17.3 ± 5.0	17.6 ± 5.1
Weight Stigma Time of Life Questionnaire†			
Onset of weight stigma			
Childhood (ages 10 and under)	3,779 (20.1)	2,126 (20.0)	1,653 (20.2)
Adolescence (ages 11–19)	4,910 (26.2)	2,816 (26.6)	2,094 (25.7)
Young adulthood (ages 20–39)	2,052 (10.9)	1,179 (11.1)	873 (10.7)
Middle adulthood (ages 40–59)	1,017 (5.4)	668 (6.3)	349 (4.3)
Older adulthood (ages 60 and up)	77 (0.4)	45 (0.4)	32 (0.4)
Never	77 (0.4)	48 (0.5)	29 (0.4)
Frequency/Distress			
Childhood	5.2 ± 1.5/5.9 ± 1.2	5.2 ± 1.6/5.9 ± 1.3	5.1 ± 1.5/5.9 ± 1.2
Adolescence	4.8 ± 1.6/5.7 ± 1.3	4.9 ± 1.6/5.7 ± 1.4	4.7 ± 1.6/5.6 ± 1.3
Young adulthood	3.6 ± 1.6/4.8 ± 1.6	3.7 ± 1.7/4.8 ± 1.6	3.5 ± 1.6/4.7 ± 1.6
Middle adulthood	3.1 ± 1.7/4.2 ± 1.7	3.1 ± 1.7/4.2 ± 1.7	2.9 ± 1.6/4.2 ± 1.7
Older adulthood	2.4 ± 1.6/3.7 ± 1.8	2.4 ± 1.6/3.8 ± 1.8	2.3 ± 1.5/3.7 ± 1.8
Last year	2.4 ± 1.5/4.2 ± 1.7	2.4 ± 1.5/4.2 ± 1.7	2.4 ± 1.5/4.2 ± 1.7
Sources of stigma†			
Family of origin	9,508 (50.7)	5,514 (52.0)	3,994 (48.9)
Extended family	6,869 (36.6)	3,940 (37.1)	2,929 (35.9)
Family of procreation	7,081 (37.7)	4,179 (39.4)	2,902 (35.6)
Friends	7,784 (41.5)	4,513 (42.6)	3,271 (40.1)
Workplace	7,688 (41.0)	4,579 (43.2)	3,109 (38.1)
School	9,347 (49.8)	5,343 (50.4)	4,004 (49.1)
Health care	8,671 (46.2)	5,086 (48.0)	3,585 (43.9)
Community	9,004 (48.0)	5,266 (49.7)	3,738 (45.8)

† Weight Stigma Time of Life Questionnaire and Interpersonal Sources of Stigma items were only administered to participants who reported experiencing weight stigma (*n* = 11,924 for total sample, *n* = 6,893 for Digital + Studio and *n* = 5,031 for Digital members). Denominators in the percentages for WSTOLQ and Interpersonal Sources of Stigma items represent the total sample size (and sample sizes for all participants with both types of WW membership).

### Correlates of weight bias internalization

Tables 3 and 4 present the participant characteristics associated with WBIS‐M and WSSQ total scores, respectively. Across both measures, WBI scores were higher among members who were younger and female, had higher BMIs, and had a younger age of overweight onset. WBI was lower in participants who were Black, were widowed or had a romantic partner, and had postgraduate education. Overall, participant characteristics accounted for 18.3% of the adjusted variance in both WBIS‐M and WSSQ scores.

**Table 3:** Demographic predictors of WBIS‐M scores in total sample

Correlations				
Variable	Body mass index	Age	Age of overweight onset	
WBIS‐M	0.36***	−0.21***	−0.22***	
Analyses of variance				
Variable	Mean ± SD	*F*	*η*_*p*_^2^	*p*
Sex		60.70	0.003	<0.001
Male	3.9 ± 1.4			
Female	4.3 ± 1.4			
Race/Ethnicity		17.46	0.004	<0.001
White, non‐Hispanic/Latino	4.3 ± 1.4			
Black or African–American	3.8 ± 1.5			
Asian or Pacific Islander	4.3 ± 1.4			
Latino, Hispanic or Mexican–American	4.3 ± 1.5			
Other	4.3 ± 1.5			
Education		10.69	0.003	<0.001
Less than high school/GED	4.6 ± 1.6			
High school/GED	4.3 ± 1.5			
Vocational/technical school (2 years)	4.4 ± 1.5			
Some college	4.3 ± 1.5			
College graduate	4.3 ± 1.4			
Postgraduate degree or higher	4.2 ± 1.4			
Marital status		82.23	0.017	<0.001
Married	4.2 ± 1.4			
Divorced	4.4 ± 1.5			
Separated	4.7 ± 1.5			
Widowed	4.0 ± 1.4			
Never married	4.7 ± 1.4			
Current significant other		143.19	0.008	<0.001
Yes	4.2 ± 1.4			
No	4.5 ± 1.5			
Body mass index category (kg m^−2^)		853.39	0.120	<0.001
<18.5	3.1 ± 1.6			
18.5–24.9	3.3 ± 1.3			
25–29.9	3.9 ± 1.3			
≥30	4.7 ± 1.3			
WW membership		6.96	<0.001	0.008
Digital + Studio	4.2 ± 1.4			
Digital	4.3 ± 1.4			
Time in WW		23.90	0.004	<0.001
3–6 months	4.4 ± 1.4			
6–12 months	4.3 ± 1.4			
1–5 years	4.2 ± 1.4			
6+ years	4.2 ± 1.4			

*** *p* < 0.001.

GED, General Education Development; SD, standard deviation; WBIS‐M, Weight Bias Internalization Scale – Modified.

**Table 4:** Demographic predictors of WSSQ total scores

Correlations				
Variable	Body mass index	Age	Age of overweight onset	
WSSQ total score	0.34***	−0.18***	−0.30**	
Analyses of variance				
Variable	Mean ± SD	*F*	*η*_*p*_^2^	*p*
Sex		27.75	0.001	<0.001
Male	33.6 ± 9.7			
Female	35.3 ± 9.7			
Race/Ethnicity		26.77	0.006	<0.001
White, non‐Hispanic/Latino	35.3 ± 9.7			
Black or African–American	31.2 ± 10.2			
Asian or Pacific Islander	35.1 ± 10.3			
Latino, Hispanic or Mexican–American	35.2 ± 9.9			
Other	35.7 ± 9.5			
Education		4.43	0.001	<0.001
Less than high school/GED	35.9 ± 9.6			
High school/GED	35.1 ± 10.1			
Vocational/technical school (2 years)	36.1 ± 9.9			
Some college	35.5 ± 10.0			
College graduate	35.3 ± 9.4			
Postgraduate degree or higher	34.8 ± 9.6			
Marital status		39.29	0.008	<0.001
Married	34.9 ± 9.6			
Divorced	35.1 ± 10.0			
Separated	36.6 ± 9.7			
Widowed	33.1 ± 10.0			
Never married	37.1 ± 9.5			
Current significant other		21.30	0.001	<0.001
Yes	35.1 ± 9.6			
No	35.9 ± 9.8			
Body mass index category (kg m^−^2)		647.66	0.094	<0.001
<18.5	29.1 ± 11.2			
18.5–24.9	29.9 ± 9.1			
25–29.9	33.1 ± 9.1			
≥30	37.8 ± 9.3			
WW membership		0.001	<0.001	0.979
Digital + Studio	35.3 ± 9.6			
Digital	35.2 ± 9.8			
Time in WW		3.60	0.001	0.013
3–6 months	35.6 ± 9.6			
6–12 months	35.2 ± 9.9			
1–5 years	35.2 ± 9.5			
6+ years	34.7 ± 9.7			

** *p* < 0.01.

*** *p* < 0.001.

GED, General Education Development; SD, standard deviation; WSSQ, Weight Self‐Stigma Questionnaire.

#### Weight stigma and WBIS‐M

When all participant characteristics with independent associations with WBIS‐M scores were included in the same regression model, significant associations were found between WBIS‐M scores and age, sex, race (Black, relative to White), BMI, marital status (divorced, separated, and never married, relative to married), age of overweight onset, and WW membership duration (6–12 months and 1–5 years, relative to 3–6 months). Table 5 shows the effects of all weight stigma variables on WBIS‐M scores, controlling for significant participant characteristics. Any experience of weight stigma was associated with significantly higher levels of WBI than not experiencing weight stigma. Experiencing weight stigma for the first time in childhood and young adulthood was associated with higher current levels of WBI, while onset of weight stigma during adolescence was associated with lower levels of WBI. Distress caused by weight stigma in the past year had a stronger association with WBIS‐M scores than did distress during other times, and generally, distress during any time period was a stronger correlate of WBIS‐M scores than frequency of experienced stigma. All interpersonal sources of stigma were significantly associated with higher WBIS‐M scores, although the *β* values for extended family and school sources were very small (<0.10).

**Table 5:** Regression of weight stigma variables on Weight Bias Internalization Scale – Modified

Weight stigma experiences				First experience of weight stigma				Weight stigma time of life				Sources of weight stigma			
Variable	B	SE	β	Variable	B	SE	β	Variable	B	SE	β	Variable	B	SE	β
Teasing	0.54	0.02	0.19***	Child	0.09	0.03	0.03***	Child‐F	0.04	0.02	0.04*	Fam‐Origin	0.35	0.03	0.10***
								Child‐D	0.19	0.02	0.16***	Fam‐Extend	0.25	0.03	0.09***
												Fam‐Pro	0.41	0.03	0.14***
Discrimination	0.58	0.02	0.19***	Adol	−0.14	0.03	−0.05***	Adol‐F	0.04	0.01	0.04***	Friends	0.38	0.03	0.13***
								Adol‐D	0.23	0.01	0.22***				
Unfair treatment	0.66	0.02	0.23***	YA	0.12	0.03	0.03***	YA‐F	0.05	0.01	0.05***	Workplace	0.42	0.03	0.14***
								YA‐D	0.23	0.01	0.27***				
Any	0.63	0.02	0.21***	MA	0.01	0.05	0.001	MA‐F	0.05	0.01	0.05***	School	0.22	0.03	0.06***
								MA‐D	0.24	0.02	0.32***				
				OA	0.14	0.15	0.01	OA‐F	0.04	0.02	0.04	Health care	0.43	0.03	0.14***
								OA‐D	0.24	0.02	0.32***				
								LY‐F	0.07	0.01	0.07***	Community	0.47	0.03	0.14***
								LY‐D	0.28	0.01	0.37***				

All analyses control for age, sex, race (reference group: White), education (reference: college), marital status (reference: married), body mass index, age of overweight onset, and WW membership duration (reference: 3–6 months). All continuous variables were centred at their means.

* *p* < 0.05.

** *p* < 0.01.

*** *p* ≤ 0.001.

Adol, adolescence; Child (F or D) = childhood (frequency or distress); Fam‐Extend, extended family; Fam‐Origin, family of origin; Fam‐Pro, family of procreation; LY, last year; MA, middle adulthood; OA, older adulthood; SE, standard error; YA, young adulthood.

When all three items for different types of experiences of weight stigma were included in the same model, the *R* ^2^ value increased by 0.05 (*p* < 0.001) from the model with only participant characteristics, with the full adjusted model explaining a total of 23.3% of the variance in WBIS‐M scores. The weight stigma onset variables contributed 0.003 to the *R* ^2^ value (*p* < 0.001, compared with participant characteristics alone), with the full adjusted model accounting for 12.7% of variance in WBIS‐M scores. When all frequency and distress scores for all time periods were included in the same model, the *R* ^2^ value increased by 0.23 (*p* < 0.001), and the full adjusted model explained 24.5% of the variance in WBIS‐M scores. All sources of stigma contributed 0.05 to the *R* ^2^ value (*p* < 0.001), accounting for a total of 17.6% of the variance in WBIS‐M scores.

#### Weight stigma and WSSQ

When including all significant participant characteristics in the same model, significant associations were found between WSSQ total scores and: age; sex; race (Black and Hispanic, relative to White) BMI; and age of overweight onset. Results were largely consistent with those found in the regression models for WBIS‐M scores (Table 6). Including the three variables for weight stigma experiences in the same model (in addition to participant characteristics) increased the *R* ^2^ by 0.10 (*p* < 0.001), explaining an adjusted total of 28.2% of the variance in WSSQ total scores. When all first experiences of weight stigma were in the same model, the *R* ^2^ value increased by 0.004 (*p* < 0.001), with the full adjusted model accounting for 10.5% of variance in WSSQ total scores. Frequency and distress scores increased the *R* ^2^ value by 0.19 (*p* < 0.001), with the total adjusted model explaining 16.6% of the variance in WSSQ total scores. All sources of stigma variables increased the *R* ^2^ value by 0.08 (*p* < 0.001), accounting for a total of 18.4% of the variance in WSSQ total scores.

**Table 6:** Regression of weight stigma variables on Weight Self‐Stigma Questionnaire – total

Weight stigma experiences				First experience of weight stigma				Weight stigma time of life				Sources of weight stigma			
Variable	B	SE	Β	Variable	B	SE	β	Variable	B	SE	β	Variable	B	SE	β
Teasing	5.25	0.15	0.27***	Child	0.58	0.18	0.03**	Child‐F	0.24	0.11	0.04*	Fam‐Origin	2.86	0.20	0.13***
								Child‐D	1.07	0.13	0.14***	Fam‐Extend	2.11	0.17	0.12***
												Fam‐Pro	2.86	0.16	0.16***
Discrimination	5.61	0.15	0.28***	Adol	−1.00	0.16	−0.06***	Adol‐F	0.38	0.07	0.06***	Friends	3.23	0.17	0.17***
								Adol‐D	1.31	0.08	0.20***				
Unfair treatment	6.14	0.13	0.32***	YA	1.01	0.22	0.04***	YA‐F	0.58	0.07	0.10***	Workplace	3.80	0.17	0.20***
								YA‐D	1.25	0.06	0.23***				
Any	6.14	0.15	0.31***	MA	0.28	0.32	0.01	MA‐F	0.60	0.08	0.10***	School	1.99	0.22	0.09***
								MA‐D	1.22	0.07	0.24***				
				OA	−0.24	1.00	−0.002	OA‐F	0.43	0.15	0.07**	Health care	3.18	0.18	0.16***
								OA‐D	1.17	0.12	0.24***				
								LY‐F	0.69	0.07	0.11***	Community	4.14	0.19	0.20***
								LY‐D	1.44	0.06	0.29***				

All analyses control for age, sex, race (reference group: White), education (reference: college), marital status (reference: married), BMI, age of overweight onset, and WW membership duration (reference: 3–6 months). All continuous variables were centred at their means.

* *p* < 0.05.

** *p* < 0.01.

*** *p* < 0.001.

Adol, adolescence; Child (F or D) = childhood (frequency or distress); Fam‐Extend, extended family; Fam‐Origin, family of origin; Fam‐Pro, family of procreation; LY, last year; MA, middle adulthood; OA, older adulthood; SE, standard error; YA, young adulthood.

#### Supplemental analyses

Tables S1–S4 present correlations, ANOVAs, and regression results for the WSSQ subscales. Some results were consistent across measures, such as associations with age, sex, BMI, race/ethnicity, and age of overweight onset. However, differences emerged with respect to education and romantic partner status, with which FES scores were significantly associated but not SD scores. Additionally, FES scores were higher in Digital + Studio versus Digital members, while SD scores showed the reverse pattern. Both subscales were significantly associated with weight stigma experiences and interpersonal sources, although effect sizes were generally larger for FES versus SD scores (*R* ^2^ change = 0.13–0.16 vs. 0.02–0.03). Early life onset of weight stigma and frequency of stigma in all time periods except the last year were also not significantly associated with SD scores but were significant for FES scores. Overall, FES scores were more strongly associated with independent variables than were SD scores (Tables S3 and S4).

## Discussion

In this large sample of adults enrolled in a commercial weight management programme (and the largest study of WBI to date), mean levels of WBI were relatively high, with almost 60% of participants scoring ≥4.0 on the 7‐point WBIS‐M scale. Over one‐third of participants scored one standard deviation above mean WBIS‐M scores previously documented in a general population sample of adults, in which approximately 20% endorsed particularly elevated levels of WBI (≥4.88) 8. Thus, the present treatment‐seeking sample, which represented approximately 4% of eligible respondents, reported substantially higher rates of elevated WBI compared with a large non‐treatment‐seeking sample 8. These differences may, in part, be attributable to the predominantly White and college‐educated sample characteristics, as well as potential selection bias of participants who were interested in weight stigma. Of note, some smaller studies of treatment‐seeking samples have reported mean WBIS scores comparable with levels found in this study 31, 32, and prior studies of patients with binge eating disorder or seeking bariatric surgery have documented higher mean scores 33, 34. Thus, these findings fit within the broader literature, suggesting that people seeking weight‐loss treatment may have elevated WBI in comparison with the general population 8, 33.

Across two measures, WBI was higher in women and those with younger age, higher BMI (particularly participants with obesity), and younger age of overweight onset. Consistent with prior research 7, 8, WBI was lower in Black versus White participants and in individuals with higher levels of education. Participants who were married or widowed had lower levels of WBI, while those who were divorced, separated or never married tended to have higher levels of WBI. These differences may reflect the protective nature of social support, which future research can examine more directly in relation to WBI.

Across both WBIS and WSSQ analyses, any experience of weight stigma was associated with higher levels of WBI. Among people who had experienced weight stigma, significant associations were found for the time of weight stigma onset and frequency and distress of stigmatizing experiences, as well as for interpersonal sources of weight stigma. Overall, reported frequency and distress of stigmatizing experiences across the lifespan accounted for more variance in WBI (e.g., 23% for WBIS‐M scores) than did stigma onset or sources. In comparison with reported frequency, distress scores had stronger associations with WBI, and these associations were particularly strong for more recent time periods of stigma (last year and middle/older adulthood). Thus, ongoing distress about weight stigma may have a more potent relationship with current WBI, although stigma frequency and distress at any time were independently associated with increased WBI levels. Consistent with this finding, experiencing weight stigma from school sources (which likely occurred during participants' younger years) had weaker associations with WBI than most other sources. However, onset of weight stigma later in life was not associated with WBI, whereas younger age of overweight onset was related to higher WBI. A potential cumulative effect may occur by which early exposure to weight stigma and continuing stigmatizing experiences in later years increase vulnerability to internalizing stigma. Alternatively, people with higher levels of WBI may recall greater experiences of and distress about weight stigma retrospectively. This underscores the need for longitudinal investigations of WBI and exposure to stigma over time.

An unexpected pattern emerged with respect to onset of weight stigma and WBI. While first experiences of weight stigma in childhood and early adulthood were associated with greater WBI, onset of weight stigma in adolescence was associated with lower current levels of WBI. Adolescents experience teasing, social exclusion, and other forms of peer victimization for many reasons aside from weight 35. It is possible that, if adolescents with overweight/obesity witness others experiencing teasing/bullying, they may perceive weight‐stigmatizing experiences as a normative social challenge, thus making them feel less alone than during other times in life when peer victimization is less common. Nevertheless, although weight stigma onset in adolescence was related to lower WBI, higher frequency and distress of weight stigma during adolescence was still associated with higher WBI, and there may be other adverse outcomes not reported in the current study that are associated with weight stigma onset during this time. These results should also be interpreted with caution due to the small *β* values for weight stigma onset (absolute values <0.10). Research on WBI in youth is currently limited 3, and this study was restricted to retrospective reports of weight stigma. More research is needed to clarify potential differential effects of first experiencing weight stigma during adolescence compared with other periods of life.

While some differences between the WBIS‐M and WSSQ measures emerged, the two measures were largely consistent in their associations with weight stigma variables. However, the FES and SD subscales diverged in their associations with education, romantic partner status, and several WSTOLQ items (including weight stigma onset). Of note, all differences in subscale scores by participant characteristics were small, and *β* values for weight stigma onset were <0.10. Given the secondary nature of these analyses, the observed differences could have been spurious findings despite having *p* values ≤0.001. Still, these differences could suggest that the two constructs measured by these subscales – anticipated stigma (FES) and internalized stigma (SD) – may indeed be distinct rather than synonymous or even complimentary 18. The FES subscale may pertain more to stigma consciousness or social identity threat (rather than internalized stigma), which account for awareness of having a devalued social identity (i.e. due to weight) and fear of maltreatment but do not require endorsement or self‐application of these negative societal views 36, 37. These divergences should be considered when interpreting the WSSQ total score and more broadly point to the need for development of additional WBI measurement. It may be useful to generate measurement items from patient‐centred communications with diverse groups of adults in focus groups and semi‐structured interviews 38, rather than from researchers' knowledge as has been performed in prior work 1, 6, in order to more accurately isolate WBI from other stigma processes. Given the high levels of WBI observed in the present study, and prior evidence of links between WBI and poor weight‐related health, efforts to develop additional measures of WBI should include individuals engaged in weight‐loss efforts.

This study is the first to examine the prevalence and correlates of WBI in a large treatment‐seeking sample and represents the largest study of WBI to date. The study's inclusion and systematic testing of multiple WBI measures within the study is novel and highlights the need to further refine assessment tools and better define the ‘phenotype’ of someone who internalizes weight bias. This study is one of few to have attempted to build a phenotype by identifying stigma‐related predictors of WBI 13, 39. The inclusion of commercial weight management programme members – including those who use only the digital plan – increases the ecological validity of our findings more than smaller studies that examine participants in tightly controlled weight‐loss trials with specific exclusion/inclusion criteria. Our findings suggest that WBI may be highly relevant to people seeking weight loss, and targeted interventions for WBI should be developed and tested in both commercial and non‐commercial weight management programmes.

However, this sample was limited in its gender and racial/ethnic diversity, thus limiting the generalizability of the findings. Prior work has found that WBI and its effects on weight loss may be stronger in White versus minority adults 15, so more research is needed to explore weight stigma and its internalization among minorities, including potentially culture‐specific aspects of WBI that may not be incorporated in current measures. In addition, the study response rate was very low, and the survey may have attracted participants for whom weight stigma was particularly relevant or salient. Thus, these findings may not be representative of WW members or people who seek weight‐loss treatment. Different survey methods (e.g., 1,000 consecutive enrollees) are needed to determine the prevalence of WBI in WW and other treatment‐seeking samples. The regression models only accounted for, at most, 24–28% of the variance in WBI. Factors not examined in this study – such as family weight history or depression – may also determine an individual's level of WBI 13, 40. Qualitative research may help to identify other aspects of treatment‐seeking adults' lived experiences that should be included in the WBI phenotype. Weight stigma variables relied on retrospective self‐report, which may be influenced by current WBI, and conclusions about causality are precluded without experimental and longitudinal evidence.

## Conclusion

Weight bias internalization was highly prevalent in a large sample of self‐selected adults enrolled in a commercial weight management programme. Above and beyond demographic correlates of WBI, experiencing weight stigma was associated with higher levels of WBI. Early onset of overweight and weight stigma, distress due to weight stigma in recent years, and a range of interpersonal sources of weight stigma were associated with greater WBI. Given the relevancy of WBI to treatment‐seeking populations, and its known associations with adverse health behaviours and outcomes 3, future studies should investigate potential impairment in weight management among those who self‐stigmatize because of weight and develop interventions to mitigate these effects.

## Funding

This study was funded by a grant from WW (the new Weight Watchers) to the University of Connecticut on behalf of R. M. P. R. L. P. is supported in part by a Mentored Patient‐Oriented Research Career Development Award from the National Heart, Lung, and Blood Institute/NIH (K23HL140176).

## Conflict of Interest Statement

R. L. P. has served as a consultant for Novo Nordisk and WW and currently receives grant support, outside of the current study, from WW. T. A. W. serves on the scientific advisory boards for WW and Novo Nordisk and receives grant support, outside of the current study, from Novo Nordisk. A. C. W. and G. D. F. are employees and shareholders of WW.

## Supporting information

Table S1. Demographic predictors of WSSQ Fear of Enacted Stigma Subscale
Table S2. Demographic predictors of WSSQ Self‐Devaluation Subscale
Table S3. Regression of weight stigma variables on Weight Self‐Stigma Questionnaire Fear of Enacted Stigma Subscale
Table S4. Regression of weight stigma variables on Weight Self‐Stigma Questionnaire Self‐Devaluation Subscale
