# Supplementary material for: Weight bias internalization in a commercial weight management sample: prevalence and correlates
Source: Obes Sci Pract. 2019 Jul 11;5(4):342–53. doi: 10.1002/osp4.354 (PMC6700514; doi:10.1002/osp4.354)
Supplement: Supplementary file 1 — Table S1. Demographic predictors of WSSQ Fear of Enacted Stigma Subscale Table S2. Demographic predictors of WSSQ Self‐Devaluation Subscale Table S3. Regression of weight stigma variables on Weight Self‐Stigma Questionnaire Fear of Enacted Stigma Subscale Table S4. Regression of weight stigma variables on Weight Self‐Stigma Questionnaire Self‐Devaluation Subscale [file OSP4-5-342-s001.docx]

**Online Supporting Information**

Supplementary Tables 1-4 summarize participant characteristics associated with the Weight Self-Stigma Questionnaire subscales of Fear of Enacted Stigma (S1) and Self-Devaluation (S2), and the weight stigma variables associated with both subscales (S3 and S4).

Table S1. Demographic predictors of WSSQ Fear of Enacted Stigma Subscale

| Correlations |  |  |  |  |
| --- | --- | --- | --- | --- |
| Variable | Body mass index | Age | Age of overweight onset | |
| WSSQ Fear of Enacted Stigma Subscale | 0.38*** | -0.20*** | -0.34*** | |
| Analyses of Variance |  |  |  |  |
| Variable | Mean ± SD | *F* | Partial eta^2^ | *p* |
| Sex |  | 24.61 | 0.001 | <0.001 |
| Male | 16.9±5.8 |  |  |  |
| Female | 17.9±5.7 |  |  |  |
| Race/Ethnicity |  | 20.43 | 0.004 | <0.001 |
| White, non-Hispanic/Latino | 17.9±5.7 |  |  |  |
| Black or African American | 15.8±6.0 |  |  |  |
| Asian or Pacific Islander | 17.4±6.2 |  |  |  |
| Latino, Hispanic, or Mexican-American | 17.7±6.0 |  |  |  |
| Other | 18.3±5.8 |  |  |  |
| Education |  | 7.72 | 0.002 | <0.001 |
| Less than high school/GED | 18.8±6.2 |  |  |  |
| High school/GED | 17.6±5.9 |  |  |  |
| Vocational/technical school (2 years) | 18.3±5.7 |  |  |  |
| Some college | 18.1±5.8 |  |  |  |
| College graduate | 17.9±5.6 |  |  |  |
| Postgraduate degree or higher | 17.5±5.8 |  |  |  |
| Marital Status |  | 72.71 | 0.015 | <0.001 |
| Married | 17.5±5.7 |  |  |  |
| Divorced | 17.9±5.9 |  |  |  |
| Separated | 19.0±6.0 |  |  |  |
| Widowed | 16.5±5.7 |  |  |  |
| Never married | 19.4±5.6 |  |  |  |
| Current significant other |  | 78.66 | 0.004 | <0.001 |
| Yes | 17.6±5.7 |  |  |  |
| No | 18.6±5.8 |  |  |  |
| Body mass index category |  | 774.75 | 0.110 | <0.001 |
| < 18.5 kg/m^2^ | 14.5±6.6 |  |  |  |
| 18.5 - 24.9 kg/m^2^ | 14.6±5.3 |  |  |  |
| 25 - 29.9 kg/m^2^ | 16.3±5.4 |  |  |  |
| ≥ 30 kg/m^2^ | 19.5±5.5 |  |  |  |
| WW Membership |  | 14.52 | 0.001 | <0.001 |
| Digital + Studio | 18.0±5.7 |  |  |  |
| Digital | 17.6±5.8 |  |  |  |
| Time in WW |  | 4.39 | 0.001 | 0.004 |
| 3-6 months | 17.9±5.7 |  |  |  |
| 6-12 months | 17.9±5.8 |  |  |  |
| 1-5 years | 17.8±5.7 |  |  |  |
| 6+ years | 17.4±5.7 |  |  |  |

Note. WSSQ = Weight Self-Stigma Questionnaire. ****p*<0.001

Table S2. Demographic predictors of WSSQ Self-Devaluation Subscale

| Correlations |  |  |  |  |
| --- | --- | --- | --- | --- |
| Variable | Body mass index | Age | Age of overweight onset | |
| WSSQ Self-Devaluation Subscale | 0.22*** | -0.13*** | -0.19*** | |
| Analyses of Variance |  |  |  |  |
| Variable | Mean ± SD | *F* | Partial eta^2^ | *p* |
| Sex |  | 20.03 | 0.001 | <0.001 |
| Male | 16.7±5.0 |  |  |  |
| Female | 17.4±5.0 |  |  |  |
| Race/Ethnicity |  | 24.05 | 0.005 | <0.001 |
| White, non-Hispanic/Latino | 17.5±5.0 |  |  |  |
| Black or African American | 15.4±5.4 |  |  |  |
| Asian or Pacific Islander | 17.7±5.4 |  |  |  |
| Latino, Hispanic, or Mexican-American | 17.5±5.1 |  |  |  |
| Other | 17.3±5.0 |  |  |  |
| Education |  | 1.46 | <0.001 | 0.20 |
| Less than high school/GED | 17.1±4.6 |  |  |  |
| High school/GED | 17.5±5.3 |  |  |  |
| Vocational/technical school (2 years) | 17.8±5.2 |  |  |  |
| Some college | 17.4±5.2 |  |  |  |
| College graduate | 17.4±4.9 |  |  |  |
| Postgraduate degree or higher | 17.3±5.0 |  |  |  |
| Marital Status |  | 7.97 | 0.002 | <0.001 |
| Married | 17.4±5.0 |  |  |  |
| Divorced | 17.2±5.1 |  |  |  |
| Separated | 17.6±5.1 |  |  |  |
| Widowed | 16.6±5.4 |  |  |  |
| Never married | 17.7±5.0 |  |  |  |
| Current significant other |  | 1.59 | <0.001 | 0.21 |
| Yes | 17.4±5.0 |  |  |  |
| No | 17.3±5.1 |  |  |  |
| Body mass index category |  | 293.26 | 0.045 | <0.001 |
| < 18.5 kg/m^2^ | 14.7±5.9 |  |  |  |
| 18.5 - 24.9 kg/m^2^ | 15.3±4.9 |  |  |  |
| 25 - 29.9 kg/m^2^ | 16.7±4.9 |  |  |  |
| ≥ 30 kg/m^2^ | 18.3±5.0 |  |  |  |
| WW Membership |  | 19.43 | 0.001 | <0.001 |
| Digital + Studio | 17.3±5.0 |  |  |  |
| Digital | 17.6±5.1 |  |  |  |
| Time in WW |  | 5.53 | 0.001 | 0.001 |
| 3-6 months | 17.7±5.0 |  |  |  |
| 6-12 months | 17.3±5.1 |  |  |  |
| 1-5 years | 17.4±5.0 |  |  |  |
| 6+ years | 17.3±5.1 |  |  |  |

Note. WSSQ = Weight Self-Stigma Questionnaire. ****p*<0.001

Table S3. Regression of weight stigma variables on Weight Self-Stigma Questionnaire Fear of Enacted Stigma Subscale

| Weight Stigma Experiences | | | | First Experience of Weight Stigma | | | | Weight Stigma Time of Life | | | | Sources of Weight Stigma | | | |
| --- | --- | --- | --- | --- | --- | --- | --- | --- | --- | --- | --- | --- | --- | --- | --- |
| Variable | *B* | *SE* | *β* | Variable | *B* | *SE* | *β* | Variable | *B* | *SE* | *β* | Variable | *B* | *SE* | *β* |
| Teasing | 3.76 | 0.08 | 0.32*** | Child | 0.44 | 0.10 | 0.04*** | Child-F | 0.25 | 0.06 | 0.07*** | Fam-Origin | 1.72 | 0.11 | 0.13*** |
|  |  |  |  |  |  |  |  | Child-D | 0.65 | 0.07 | 0.15*** | Fam-Extend | 1.51 | 0.09 | 0.14*** |
|  |  |  |  |  |  |  |  |  |  |  |  | Fam-Pro | 1.60 | 0.09 | 0.15*** |
| Discrimination | 4.49 | 0.08 | 0.37*** | Adol | -0.73 | 0.09 | -0.07*** | Adol-F | 0.33 | 0.04 | 0.10*** | Friends | 2.20 | 0.10 | 0.20*** |
|  |  |  |  |  |  |  |  | Adol-D | 0.76 | 0.05 | 0.20*** |  |  |  |  |
| Unfair  Treatment | 4.50 | 0.08 | 0.38*** | YA | 0.66 | 0.12 | 0.05*** | YA-F | 0.50 | 0.04 | 0.15*** | Workplace | 2.89 | 0.09 | 0.27*** |
|  |  |  |  |  |  |  |  | YA-D | 0.69 | 0.03 | 0.22*** |  |  |  |  |
| Any | 4.33 | 0.08 | 0.36*** | MA | 0.34 | 0.18 | 0.02 | MA-F | 0.54 | 0.04 | 0.16*** | School | 1.42 | 0.12 | 0.11*** |
|  |  |  |  |  |  |  |  | MA-D | 0.67 | 0.04 | 0.23*** |  |  |  |  |
|  |  |  |  | OA | -0.07 | 0.56 | -0.001 | OA-F | 0.38 | 0.08 | 0.11*** | Health Care | 2.37 | 0.10 | 0.20*** |
|  |  |  |  |  |  |  |  | OA-D | 0.65 | 0.07 | 0.24*** |  |  |  |  |
|  |  |  |  |  |  |  |  | LY-F | 0.53 | 0.04 | 0.15*** | Community | 3.27 | 0.10 | 0.27*** |
|  |  |  |  |  |  |  |  | LY-D | 0.80 | 0.03 | 0.29*** |  |  |  |  |

Note. ****p*<0.001

All analyses control for age, sex, race (reference group: white), education (reference: college), marital status (reference: married), BMI, age of overweight onset, and WW membership type and duration (reference: 3-6 months). All continuous variables were centered at their means. Child (F or D) = Childhood (frequency or distress); Adol = Adolescence; YA = Young Adulthood; MA = Middle Adulthood; OA = Older Adulthood; LY = Last Year. Fam-Origin = Family of origin; Fam-Extend = Extended family; Fam-Pro = Family of procreation. Participant characteristics accounted for 22.7% of the variance in Fear of Enacted Stigma scores. All three weight bias experiences items contributed to an *R^2^* change of 0.16 (*p*<0.001, total adjusted *R^2^*=0.39). All time periods for first experiences of weight stigma contribute to an *R^2^* change of 0.007 (*p*<0.001, total adjusted *R^2^*=0.13). When all weight stigma frequency/distress items were included in the same model, the *R^2^* value increased by 0.22 (*p*<0.001, total adjusted *R^2^*=0.21). When all sources of stigma variables were included in the same model, the *R^2^* changed by 0.13 (*p*<0.001, total adjusted *R^2^* = 0.25).

Table S4. Regression of weight stigma variables on Weight Self-Stigma Questionnaire Self-Devaluation Subscale

| Weight Stigma Experiences | | | | First Experience of Weight Stigma | | | | Weight Stigma Time of Life | | | | Sources of Weight Stigma | | | |
| --- | --- | --- | --- | --- | --- | --- | --- | --- | --- | --- | --- | --- | --- | --- | --- |
| Variable | *B* | *SE* | *β* | Variable | *B* | *SE* | *β* | Variable | *B* | *SE* | *β* | Variable | *B* | *SE* | *β* |
| Teasing | 1.50 | 0.08 | 0.15*** | Child | 0.02 | 0.11 | 0.01 | Child-F | -0.001 | 0.06 | <0.001 | Fam-Origin | 1.12 | 0.11 | 0.09*** |
|  |  |  |  |  |  |  |  | Child-D | 0.42 | 0.08 | 0.10*** | Fam-Extend | 0.60 | 0.10 | 0.06*** |
|  |  |  |  |  |  |  |  |  |  |  |  | Fam-Pro | 1.28 | 0.09 | 0.13*** |
| Discrimination | 1.12 | 0.08 | 0.11*** | Adol | -0.27 | 0.09 | -0.03** | Adol-F | 0.05 | 0.04 | 0.02 | Friends | 1.03 | 0.10 | 0.10*** |
|  |  |  |  |  |  |  |  | Adol-D | 0.55 | 0.05 | 0.15*** |  |  |  |  |
| Unfair  Treatment | 1.38 | 0.08 | 0.14*** | YA | 0.35 | 0.12 | 0.03** | YA-F | 0.09 | 0.04 | 0.03* | Workplace | 0.93 | 0.10 | 0.09*** |
|  |  |  |  |  |  |  |  | YA-D | 0.55 | 0.04 | 0.18*** |  |  |  |  |
| Any | 1.65 | 0.08 | 0.16*** | MA | -0.03 | 0.18 | -0.001 | MA-F | 0.08 | 0.05 | 0.02 | School | 0.56 | 0.13 | 0.05*** |
|  |  |  |  |  |  |  |  | MA-D | 0.55 | 0.04 | 0.19*** |  |  |  |  |
|  |  |  |  | OA | -0.23 | 0.57 | -0.004 | OA-F | 0.07 | 0.09 | 0.02 | Health Care | 0.81 | 0.10 | 0.07*** |
|  |  |  |  |  |  |  |  | OA-D | 0.52 | 0.07 | 0.19*** |  |  |  |  |
|  |  |  |  |  |  |  |  | LY-F | 0.16 | 0.05 | 0.04*** | Community | 0.86 | 0.11 | 0.07*** |
|  |  |  |  |  |  |  |  | LY-D | 0.64 | 0.04 | 0.23*** |  |  |  |  |

Note. **p*<0.05 ***p*<0.01 ****p*<0.001

All analyses control for age, sex, race (reference group: white), marital status (reference: married), BMI, age of overweight onset, and WW membership type and duration (reference: 3-6 months) (education was not included due to non-significant independent effects). All continuous variables were centered at their means. Child (F or D) = Childhood (frequency or distress); Adol = Adolescence; YA = Young Adulthood; MA = Middle Adulthood; OA = Older Adulthood; LY = Last Year. Fam-Origin = Family of origin; Fam-Extend = Extended family; Fam-Pro = Family of procreation. Participant characteristics accounted for 8.3% of the variance in Self-Devaluation scores. When all three weight bias experience items were included in the same model, all were significant (*p*<0.03), and the *R^2^* value increased by 0.02 (*p*<0.001, total adjusted *R^2^*=0.10). When all first stigma experiences were included in one model, the *R^2^* increased by 0.001 (*p*=0.029, total adjusted *R^2^*=0.05). When all frequency/distress items were included in the same model, the *R^2^* value increased by 0.11, *p*<0.001, total adjusted *R^2^*=0.07. When all sources of stigma items were included the same model, the *R^2^* increased by 0.03, *p*<0.001, total adjusted *R^2^*=0.07.
